# Nutritional Deficiencies and Reduced Bone Mineralization in Ulcerative Colitis

**DOI:** 10.3390/jcm14093202

**Published:** 2025-05-06

**Authors:** Filippo Vernia, Emanuela Ribichini, Giorgia Burrelli Scotti, Giovanni Latella

**Affiliations:** 1 Department of Life, Health, and Environmental Sciences, Division of Gastroenterology, Hepatology, and Nutrition, University of L’Aquila, Piazza S. Tommasi, 1-Coppito, 67100 L’Aquila, Italy; giovanni.latella@univaq.it; 2 Department of Translational and Precision Medicine, Sapienza University, 00185 Rome, Italy; emanuela.ribichini@uniroma1.it (E.R.); giorgia.burrelliscotti@uniroma1.it (G.B.S.)

**Keywords:** ulcerative colitis, UC, inflammatory bowel diseases, IBD, vitamin D, vitamin K, calcium, diet, nutrition, deficiency

## Abstract

**Background:** Inadequate dietary intake of vitamin D, vitamin K, and calcium, as well as sub-optimal sunlight exposure, can lead to bone loss in the general population, and more so in patients with ulcerative colitis, who are burdened by additional predisposing factors for osteoporosis, such as chronic inflammation and cortisone use. However, micronutrient deficiencies, if present, are easily corrected by nutritional intervention. While the relation between calcium and vitamin D and bone metabolism is well known, fewer data are available for vitamin K, for both healthy individuals and patients. The aim of this review is to provide an overview of recent reports focusing on nutritional deficits relevant to the development of osteoporosis/osteopenia in patients affected by ulcerative colitis. **Methods:** A systematic electronic search of the English literature up to January 2025 was performed using Medline and the Cochrane Library. **Results:** Despite being central in bone mineralization, data on dietary calcium intake in ulcerative colitis are relatively scarce, deriving mostly from mixed inflammatory bowel disease cohorts. Although lower than controls, dietary calcium intake approaches the recommended daily allowance, which establishes the necessary daily intake of nutrients. Conversely, vitamin D and vitamin K deficiencies are highly prevalent in ulcerative colitis patients. The widely shared opinion that milk and lactose-containing foods, as well as vegetables, worsen diarrhea is a prime determinant of inadequate vitamin D and vitamin K intake. **Conclusions:** Increased awareness of the importance of nutrition and the common occurrence of nutritional deficits represents the first step for the development of dietary intervention strategies to counteract the increased risk of osteoporosis in ulcerative colitis patients.

## 1. Introduction

**Figure 1:**
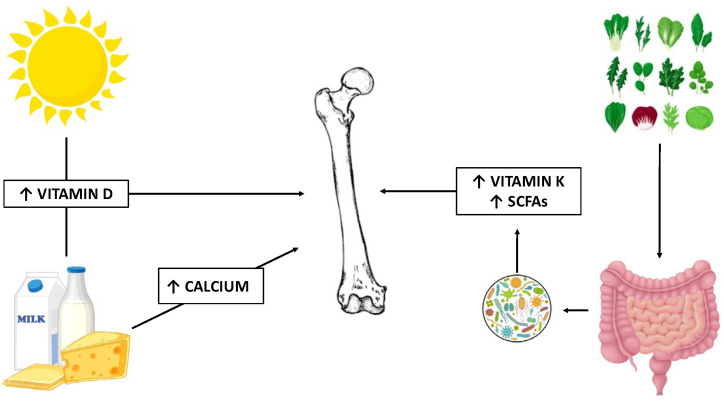
**Main modifiable determinants of bone health**. Sunlight exposure represents the main determinant of active vitamin D in the presence of diets rich in dairy products, providing the recommended daily allowances of vitamin D and calcium. Microbial metabolism of dietary green leafy vegetables provides metabolically active compounds, including vitamin K and SCFAs, that exert positive effects on the bone. SCFAs: short-chain fatty acids.

Increased risk for osteopenia and osteoporosis is well documented in ulcerative colitis (UC) and represents a leading cause of morbidity [1]. Age-related bone resorption is the most relevant factor leading to reduced bone mineral density (BMD) in healthy subjects, and more so in postmenopausal women [2]. Several additional mechanisms affect bone wellbeing in UC, including chronic inflammation and high levels of pro-inflammatory cytokines, repeated or prolonged courses of corticosteroids [3,4], and nutritional deficits of critical micronutrients [5,6,7]. Alterations of intestinal microbiota, abnormal concentrations of bacterial metabolic products, and disruption of the intestinal barrier also affect the gut–bone axis in inflammatory bowel disease (IBD) and contribute to or increase the risk of osteoporosis and osteopenia [8,9,10] (Figure 1). Reduced physical activity is an additional risk factor for reduced BMD in IBD patients [11].

The complex interplay between the different mechanisms leading to osteoporosis is still to be elucidated, as nutritional deficits, dysregulated interaction between the bone and the immunologic system, hormonal control, and the gut microbiota system are involved to a variable extent in individual patients.

This review is focused on the role of nutrition and micronutrients, including vitamin D, vitamin K, and calcium, in the genesis or worsening of osteoporosis and osteopenia in ulcerative colitis and suggests possible intervention.

## 2. Materials and Methods

A systematic electronic search of the English literature up to January 2025 was performed using Medline and the Cochrane Library. The search strategy used a combination of Medical Subject Headings (MeSH) and keywords, as follows: “vitamin D”, “vitamin D deficiency”, “vitamin K”, “vitamin D deficiency”, “calcium”, “calcium deficiency”, “micronutrient deficiency”, “diet”, “ulcerative colitis”, “UC”, “Inflammatory Bowel Disease”, “IBD”, “osteoporosis”, “osteopenia”, “bone”, “bone mineral density”, “fractures”.

Three authors selected relevant studies by screening the abstracts. Additional references were included after a review of the bibliography of the identified papers and review articles. Any difference was resolved by consensus, referring to the original articles.

Out of 763 citations, 150 relevant articles were selected and included in the present narrative review.

## 3. Results

### 3.1. Osteopenia, Osteoporosis, and Risk of Fractures in IBD: Focus on UC

Osteopenia and osteoporosis are common in patients with UC, and the decrease in BMD usually involves the entire skeleton [1]. The prevalence of these conditions varies in different cohorts, ranging from 22 to 77% and from 17 to 41%, respectively, in studies published in the last 30 years [1,12,13,14].

Risk increases with disease duration, and bone fractures occur in up to 40% of UC patients at 25-year follow-up [15,16]. The reduced BMD involves the entire skeleton, similarly to rheumatoid arthritis, in which bone loss is not limited to the small joints, where inflammation is most frequent [1,17].

In historic cohorts, a 40% higher incidence of bone fracture was reported in IBD patients than in the general population (86.2 per 10,000 in Crohn’s disease (CD) and 112.4 per 10,000 in UC) [18]. This was confirmed by the Danish registry, which reported an incidence of osteoporosis twice as high in patients with IBD compared to controls [19].

Chronic inflammation is considered a primary pathogenetic factor in UC, favoring the imbalance of the receptor activator of nuclear factor kB ligand (RANKL)/osteoprotegerin (OPG) system and bone resorption. Moreover, inflammation induces the redistribution of many crucial micronutrients from circulation to other organs, reducing bioavailability [20,21]. This effect is likely mediated by proinflammatory cytokines that suppress the hepatic production of many carrier proteins, increase the capillary permeability (favoring the leak of albumin into the extravascular space), and promote the sequestration of some micronutrients into the liver [20,21].

However, the introduction of biologics aimed at suppressing disease activity and chronic inflammation did not induce relevant changes in the prevalence of osteoporosis. In 2019, the Spanish registry reported metabolic bone disease in 66.4% and osteoporosis in 21.4% of IBD patients [22]. These figures are comparable to those reported in previous decades. Similarly, the incidence of osteoporosis (14.2%) in a recent Danish study including 513 patients did not differ from that in different cohorts investigated in the pre-biologic era [19]. Conversely, the Swiss registry suggests that early anti-TNF-α treatment (<24 months after diagnosis) significantly reduces the risks of low BMD and osteoporosis after a 10-year follow-up (11.4% vs. 28.2%, *p* < 0.001) [23].

Indirect evidence on the role of inflammation is provided by UC patients undergoing colectomy. Osteopenia in UC patients ranges from 26 to 55% after ileal pouch anal anastomosis (IPAA) [24,25,26], and osteoporosis from 13 to 32% [25]. As these values are slightly lower than in unoperated patients, surgery, to some extent, improves BMD. Removal of the target organ and turning off systemic inflammation are likely involved, but discontinuation of corticosteroids and improvement in nutritional status may contribute as well [26,27,28]. The relative weight of different mechanisms is still unsettled.

High-dose corticosteroids impair the RANK/RANKL/OPG balance, favoring bone resorption and negatively affecting its reconstitution [29]. Although these effects progressively diminish over time, reduced bone formation is the main factor responsible for low BMD in longstanding corticosteroid treatment [30], outweighing the favorable effect of reducing inflammation. Despite documented effects on the ACTH–cortisol axis, suggesting systemic effects, long-term budesonide does not increase the risk of osteoporosis and osteopenia [31].

Inadequate nutritional intake or absorption and increased losses of micronutrients all result in a deficit of vitamins and minerals involved in bone wellbeing. The importance of micronutrients with antioxidant activity in IBD, including selenium, magnesium, and zinc, as well as vitamin C, retinol, and β-carotene, has recently been reviewed [32,33]. Direct antioxidant effects of micronutrients modulate chronic inflammation and influence the composition and diversity of gut microbiota [34]. Both factors exert positive effects on BMD in UC patients, in addition to the primary role of calcium, vitamin D, and vitamin K in bone homeostasis. The possibility to improve bone metabolism through nutritional intervention and the correction of micronutrient deficit represents a feasible approach [35].

### 3.2. Calcium

Despite being central in bone mineralization, data on dietary calcium intake in IBD patients are relatively scarce. A large proportion of patients believes that dietary modifications represent a tool to control abdominal symptoms. Thus, up to 68% of them limit their diet to improve their quality of life [36]. This issue is discussed in detail in the section concerning vitamin D.

As dairy products represent the main source of calcium, this implies a reduced intake of the micronutrient (mean intake 692.3 ± 405.8 mg/day) [37]. Significantly lower calcium intake in patients compared to controls (837.8 ± 482.0 SD vs. 991.0 ± 536.0 SD mg/day, *p* < 0.001) was also reported in a cohort of 187 IBD patients [38].

Overall, as reported in a recent meta-analysis, most studies report inadequate calcium intake [39]. However, despite being significantly lower than in controls, dietary calcium intake was close to the recommended daily allowance (RDA) in most series [38,40,41].

Low calcium intake correlates with reduced BMD [42,43]. Nutritional intervention and long-term calcium supplementation, however, did not improve BMD in healthy subjects [44] or in IBD patients [45,46,47].

### 3.3. Vitamin D

Vitamin D is a pro-hormone that is actively involved in the regulation of bone metabolism as well as a number of extra-skeletal functions [48,49].

Vitamin D influences bone remodeling both through indirect and direct mechanisms [50,51]. The indirect mechanisms consist of the control of intestinal calcium absorption, renal calcium reabsorption, and the modulation of parathyroid hormone (PTH) production [52,53].

Experimental studies indicate that vitamin D has direct effects on bone cells, favoring osteoblast proliferation [54,55] and survival [56,57], enabling osteoblast differentiation from immature mesenchymal stromal cells [58,59,60], and modulating osteoclastogenesis [61,62].

Studies in vitamin D receptor (VDR)-deficient mice reported that osteoblast-specific VDR deletion results in a minimal increase in trabecular bone volume [63], while osteoblast VDR overexpression results in increased bone mass due to increased osteoblastic bone formation and reduced osteoclastic resorption [64,65].

The effects of vitamin D on the immune system are related to VDR expression on immune cells, including macrophages, dendritic cells, and B- and T cells [66,67].

In the acquired immune system, vitamin D inhibits the proliferation of B- and T-cells, as well as the T-cell production of pro-inflammatory cytokines, including IL-2, interferon (IFN)-γ, IL-17, and TNF-α [68].

Conversely, vitamin D induces the production of IL-10 and other anti-inflammatory cytokines by regulatory T-cells and IL-4 by Th2 cells [68,69]. These results suggest that hypovitaminosis D might influence disease activity in IBD patients.

Indeed, vitamin D has been linked to a wide range of biological activities, including the modulation of gut mucosal immunity and the integrity of the intestinal barrier [70,71]. A direct role has also been suggested [72] in disease occurrence and disease activity in IBD patients.

Plasma levels lower than 20 ng/mL and between 21 and 29 ng/mL indicate vitamin D deficiency and insufficiency, respectively [73,74]. The prevalence of vitamin D deficiency/insufficiency is high in IBD patients, ranging from 36.7% to 51.1% in different series, according to sun exposure, dietary habits, supplementation, disease location, and race [35,75,76,77]. Low vitamin D levels result both from inadequate dietary intake and inadequate sunlight exposure, preventing the conversion of 7-dehydrocholesterol to pre-vitamin D3 and vitamin D3.

This issue is of paramount importance in elderly patients, as low vitamin D occurs in 38% and osteoporosis or osteopenia in 49% of them [78].

The mean vitamin D intake is significantly lower in UC patients than in controls, and dietary vitamin D is adequate in a minority of UC patients (13–50%) [35,79]. No significant difference in vitamin D intake was recently observed in UC vs. CD patients [35,37,80].

The widely shared opinion that milk and lactose-containing foods may worsen diarrhea is a prime determinant of inadequate calcium and vitamin D intake in UC, irrespective of documented lactose malabsorption and intolerance or of the lactose content of individual servings [38,81]. Indeed, 70% of patients admit to self-prescribed dietary restrictions, and 84% of them avoid dairy products [37]. As diarrhea is less prominent in distal colitis, patients with left-sided colitis and pancolitis restrict vitamin D intake more than those with proctitis (*p* = 0.02) [82].

Besides dietary restrictions, low sunlight exposure in patients with UC [83] represents an additional mechanism favoring low vitamin D levels.

The hypothesis that low vitamin D intake may affect disease activity is controversial. It derives from observations in a small series of patients reporting that less than 10% of patients with active disease meet the recommendation for vitamin D intake [84]. This may be clinically relevant as some studies suggest that vitamin D deficiency contributes to more aggressive disease behavior and impaired response to biological therapy in IBD patients [85,86,87].

Although low vitamin D levels, low albumin levels, and iron-deficiency anemia [88] represent red flags for aggressive disease course, supplementation did not modify the clinical course of the disease. Small retrospective studies reported an association between vitamin D levels and anti-TNFα treatment persistence [89,90,91]. Preliminary evidence suggests that the same applies to anti-integrin therapy [92]. Conversely, a prospective study carried out in a cohort of 1107 patients did not support the hypothesis [93].

Irrespective of the role of vitamin D in the response to treatment and disease control, adequate intake and blood concentrations in UC patients are of prime importance to reduce the incidence of bone disease and minimize the burden of extraintestinal problems on the quality of life of these patients.

### 3.4. Vitamin K

Vitamin K is required for the synthesis of coagulation factors. It also acts as a cofactor in the carboxylation of bone proteins, including osteocalcin [94], and is thus involved in bone wellbeing [95]. The carboxylation of glutamic acid in gamma-carboxyglutamic acid promotes the binding of calcium to these proteins and partially counteracts the risk for osteoporosis [94]. Noteworthy is that the harmful effect of a vitamin K deficit on bone metabolism is present at blood concentrations that do not affect clotting, as vitamin K is preferentially used in the synthesis of coagulation factors rather than for bone metabolism [96].

Diet represents the primary source of vitamin K1 (phylloquinones), but bacterial metabolism in the gut provides additional vitamin K2 (menaquinones). Thus, the prolonged use of antibiotics favors deficiency.

Low concentration of vitamin K in IBD patients correlates with decreased bone mineral density [97,98]. A significant inverse association between vitamin K in diet and risk of fractures (RR = 0.78, 95% CI: 0.56–0.99; I^2^ = 59.2%, *p* = 0.04) is also present [99,100].

Significantly lower vitamin K intake in IBD patients compared to controls has been recently reported [35], and more so in patients with active disease, supporting the view that vitamin K shortage may play some role in the genesis of osteoporosis in patients with UC. Low median vitamin K intake was also reported in other series of adults and children with UC [101,102], but the possible connection with clinical activity was less clear [101].

The direct measurement of vitamin K levels is difficult. Moreover, circulating levels change over short time intervals. The dosage of prothrombin time (PT), used in clinical settings as a surrogate marker of severe vitamin K deficiency, is inaccurate and underestimates the prevalence of deficiency [103]. Thus, the concentration is evaluated by measuring vitamin K-dependent carboxylation products, such as protein induced by vitamin K absence-II (PIVKA-II) and undercarboxylated osteocalcin (ucOC) [103]. These indirect markers are not routinely measured in clinical laboratories, resulting in limited high-quality data on this topic in IBD. Some studies reported significantly higher levels of PIVKA-II and ucOC in CD and UC patients [102,104,105], and a meta-analysis on fat-soluble vitamin deficits confirmed vitamin K deficiency by measuring ucOC in CD patients [86]. The same meta-analysis, however, did not evaluate vitamin K levels in UC patients due to a lack of studies [86].

Evidence from animal studies supports the role of vitamin K in bone metabolism and the advantage of supplementation. Conversely, daily supplementation with 1 mg of vitamin K over 12 months did not increase the levels of bone health markers such as bone-specific alkaline phosphatase, C-terminal telopeptide of type I collagen, and urinary N-telopeptides of type I collagen in a small series of IBD patients [106]. Similar findings were reported in the general population [107,108]. BMD (*p* = 0.21) and fracture rates (*p* = 0.62) were comparable after a 2-year follow-up in 1983 healthy subjects treated with risedronate, irrespective of the concurrent treatment with vitamin K2 (45 mg/day) [109]. However, these negative reports are biased by several confounding factors. Studies were carried out in patients with advanced osteoporosis, the follow-up period never exceeded two years, and the effectiveness of vitamin K administration was evaluated in patients undergoing concomitant effective treatment with vitamin D or risedronate. The real clinical role of vitamin K in the prevention of osteoporosis or the slowing of the progression from osteopenia to osteoporosis in healthy subjects and UC patients is still to be elucidated.

### 3.5. Microbiota, Diet, and Bone Health

The composition and diversity of the microbiota interacting with epithelial and immune cells in the gut influence critical functions of the host [34,110]. The normal intestinal microbiota is essential for effective mucosal barrier function, including mucus production, which modulates the development of the immune system, triggers the immune response, and helps to maintain non-inflammatory homeostasis in the gut [111].

Resident bacteria produce several compounds, including short-chain fatty acids (SCFAs), secondary bile acids (BAs), indole derivatives, and amines [112,113], which are relevant tools in the interaction between the intestinal microbiota and host metabolism and immune system. DNA methylation, histone modifications, and the production of microRNA are all influenced by intestinal microflora metabolites and dietary components. This results in relevant epigenetic effects through the modulation of gene expression at transcriptional and post-transcriptional levels that result in physiological and pathophysiologic processes, including inflammatory diseases [114].

SCFAs present in stool mainly consist of the C2–C4 acids acetate, propionate, and butyrate at a molar ratio of 60:25:15, respectively. SCFAs are produced by bacterial strains fermenting unabsorbable dietary carbohydrates and directly depend on food choice. The proportion of different SCFAs varies in relation to the composition of gut bacteria. Butyrate production is raised in the presence of *Firmicutes*, including *Faecalibacterium prausnitzii*, *Clostridium leptum*, *Eubacterium hallii*, and *Roseburia* spp. [115]. Butyrate is central for colonic mucosal health, representing the preferential energy substrate for colonocytes [116] and having complex systemic effects ranging from the inhibition of histone deacetylase to direct gene modulation. Conversely, when other bacteria prevail, such as *Acetobacteria*, *Acetogenia*, and *Clostridia*, acetate production is increased by splitting the C4 acid butyrate [117], and its fecal concentration is lower.

In turn, their concentration in the intraluminal environment modulates the composition of intestinal flora, providing a direct energy source to bacteria and regulating the transcription of bacterial genes and the interplay with intestinal mucosa and the immune response. Also, in this respect, the interaction between diet and flora is strict and bidirectional.

As far as the bone is concerned, the balance of bone formation and resorption is modulated primarily by butyrate and propionate. Acting on T-regs, they inhibit osteoclasts and increase bone mass, influencing PTH-induced metabolism and calcitonin [118]. Thus, SCFAs directly and indirectly affect bone density and quality through the modulation of hormones, growth factors, and cytokines involved in bone remodeling. Conversely, altered microbiota and an increased load of antigens derived from food and bacteria due to disruption of the intestinal mucosal barrier function play a significant role in the activation of intestinal immune cells and their migration to other organs. This includes the immune activation of bone marrow cells, decreasing bone formation and increasing bone resorption [119].

A similar relationship also exists between BAs and several systemic metabolic and immune effects relevant for bone wellbeing. Although BAs favor bone formation, excessively low or high BA concentrations shift the balance toward bone resorption. BAs activate the farnesoid-X-receptor and bind to several receptors [120], including the transmembrane G protein-coupled receptor 5 and the vitamin D receptor, which are crucial for normal bone cell activity and bone remodeling. Interestingly, secondary BAs resulting from bacterial metabolism have a higher affinity than primary BAs for many receptors, underlining the systemic effects of the gut microbiome. The identification of new BA conjugations resulting from specific bacterial metabolic pathways [121] will likely provide a key for identifying specific stool metabolome signatures in health and disease.

Gut bacteria directly affect nutrient balance, producing significant amounts of vitamin K, vitamin A, and B vitamins [122]. The relation is bidirectional, as diet and micronutrients (i.e., vitamins D, C, A, E, and B6) in turn affect the composition of intestinal flora. The relationship between vitamin D and microbiota has been recently reviewed by Aggeletopoulous [123,124]. High levels of vitamin D through VDR expression increase the mucosal barrier function and modulate mucosal immunity and the gut microbiota. Vitamin D and the activation of VDR, which are not expressed by bacteria, shift the composition of intestinal flora, favoring beneficial bacteria. The combination of vitamin D supplements and probiotics appears to be superior to vitamin D or probiotics alone in patients with IBD [125].

Fecal microbiota transplantation in patients with UC resulted in increased levels of vitamin D, further supporting the hypothesis that intestinal bacteria, besides ultraviolet radiation, may be important in determining the serum concentrations of vitamin D [126].

The interaction between bacteria, vitamin K2, and BMD in IBD patients has also been reported, as reduced microbiota diversity affects the levels of ucOC in CD patients [127].

Food and diet actively modulate the gut microbiota. Changes in long-term dietary patterns are responsible for about 10% of plasma metabolome variations and show several associations with serum metabolome that exceed those of genetics and fecal microbial composition [128].

Diets rich in unabsorbable carbohydrates increase the number of *Bifidobacteria* and decrease the abundance of *Clostridia* and *Bacteroidetes*.

Besides the prebiotic role of dietary fiber and complex carbohydrates, specific foods contain specific bacterial species. Fermented dairy products, formed by the addition of strains converting milk lactose into lactic acid, provide bacteria that are beneficial for human—and bone—health [129] and exert additional positive effects on bone metabolism, unrelated to the calcium and vitamin D content [130].

High-fat diets unfavorably impact microbiota, leading to an imbalance between *Firmicutes* and *Bacteroidetes*, possibly due to different bactericidal activity of BAs on these phyla [131].

Protein-rich diets also negatively affect the intestinal microbiome and enhance the production of trymethylamine-N-oxide and indoxyl sulfate, which interfere with normal bone and mineral metabolism.

The modulation of microbiota and changes in bacterial metabolites are central to the effects of different dietary patterns [132]. As different nutrients contained in a meal interact, different dietary patterns, such as the Western diet and the Mediterranean diet, favorably influence bone metabolism and reduce the risk of osteoporosis.

This applies especially to the Mediterranean diet, which is characterized by a high consumption of unsaturated fat, a high intake of vegetables, fruits, legumes, and cereals, and is high in essential nutrients and other bioactive components such as calcium and vitamin D, protein, and magnesium, which are involved in maintaining bone health [133].

The effect is in addition to that of the sheer micro- and macronutrient content. This issue is basically unexplored in UC patients despite the profound alteration of stool SCFAs and lactic acid being well documented in patients with active disease [134].

Another central point in the relation between diet and gut bacteria is breast-feeding in newborns.

Breast milk is not sterile and favors proper intestinal colonization, directly providing useful bacteria and factors, like immunoglobulins, lysozyme, and lactoferrin, that modulate the composition of the bacteria that first colonize the intestinal tract. Breast-feeding increases the number of *Bifidobacteria,* which are useful for gut and bone wellbeing. Babies that are breast-fed in the first 9 months of their lives have higher BMDs compared to those who are fed milk formula [135]. However, prolonged breast-feeding reduces the BMD of lactating mothers and subsequently increases the risk of vertebral fractures in postmenopausal age. Again, this problem remains unaddressed in UC patients.

Probiotic supplementation is the subject of ongoing research, as some researchers suggest that they exert a beneficial effect on bones through nutrient uptake and processing [136]. However, there is high heterogeneity between studies, co-interventions are often used rather than probiotics alone, the follow-up is limited to short time intervals, and strong data are lacking in UC patients [136].

### 3.6. Dietary Intervention

The patient perception of the relevance of diet in IBD patients is high, but information and dietary guidance are often inadequate or unreliable [137]. Unsupervised dietary manipulation and food avoidance often result in an inadequate dietary intake of micronutrients [138]. The unnecessary restriction of milk consumption in the absence of documented lactase deficiency is common. This attitude over time has led to a reduced consumption of milk, but not cheese, in the US population, contributing to inadequate calcium [139] and vitamin D intake in the general population. The “nocebo” effect, i.e., the negative modification of symptoms induced by an inactive substance or procedure expected to be harmful, is triggered by the fear of lactose side effects. This has been well documented in irritable bowel syndrome [140] but is also present in UC. Thus, a lactose tolerance breath test is advisable to identify those patients who do not require milk derivative restrictions and may increase the dietary intake of calcium and vitamin D. Patients with documented lactose malabsorption, in the absence of lactose intolerance (defined as symptoms induced by a lactose load during or shortly after the lactose tolerance test) [141], should be encouraged to reduce, but not terminate, lactose-containing food. The possibility to eat up to 10 g of lactose without significant symptoms has been well documented in the past in lactose malabsorbers [142]. Moreover, the regular ingestion of lactose progressively reduces abdominal symptoms in malabsorbers, possibly shifting gut microbiota by favoring β-galactosidase-producing strains [143].

Dairy products with a low lactose content, i.e., hard cheese, represent a further viable option [144], considering that milk derivatives represent the source of 50 to 65% of daily reference calcium intake in Western countries. Noteworthy is that a very low consumption of this class of food during childhood is associated with an increased risk of fractures [145].

Conversely, an excessive increase in calcium intake is neither healthy nor useful, as intake and absorption are inversely related. About half of ingested calcium is absorbed at low intakes (200 mg/day), whereas absorption does not exceed 15% when intake exceeds 2 g/day.

Another unsupported nutritional belief in UC patients is the need to restrict or abolish food rich in dietary fiber, which is more so if diarrhea is present. This results from finding undigested fiber and intact vegetables in loose stools and the postulation that they worsen diarrhea. Conversely, faster intestinal transit time does not allow complete bacterial metabolization of unabsorbed carbohydrates and lessens the production of useful SCFAs [146]. Reasonable amounts of fiber in the diet of UC patients, including green leafy vegetables, imply a higher availability of SCFAs, with favorable effects on intestinal symptoms [147], besides increasing the intake of vitamin K.

## 4. Conclusions

Adequate serum vitamin D levels are related to human health. Despite a huge number of experimental and human studies documenting an increased susceptibility to diseases ranging from osteoporosis to autoimmune diseases, inflammation, infections, and cancer, the real role of vitamin D supplementation is still to be defined. Moreover, the most appropriate dosage required in different populations and patient groups, including ulcerative colitis patients, has not yet been established. What constitutes an adequate intake of vitamin K is even less well defined, resulting in different nutritional guidelines in different countries.

**Figure 2:**
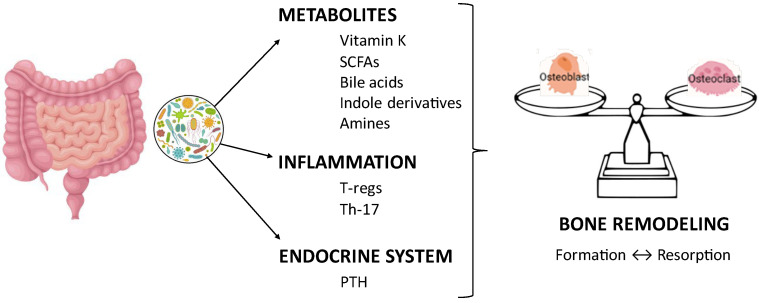
**Interactions between gut microbiome and bone remodeling.** Gut microbiome directly regulates bone remodeling through the production of active metabolites, such as vitamin K, SCFAs, bile acids, indole derivatives, and amines. Indirect effects include SCFA-mediated immune regulation, which inhibits osteoclasts and increases bone mass, as well as PTH and calcitonin regulation. SCFAs: short-chain fatty acids; PTH: parathormone.

In patients with ulcerative colitis, more so for those with inactive or mildly active disease, dietary intervention may prove to be adequate in providing enough calcium, vitamin D, and K to favor bone health and minimize the risk of osteopenia and osteoporosis. Additional advantages may reside in the anti-inflammatory activity of vitamins D and K [148]. This is easily attained by avoiding the unnecessary restriction of dietary fiber, milk, and milk derivatives. Favoring the consumption of low-lactose cheese and lactose-free foods is helpful in patients with severe lactase deficiency. In addition to micronutrients, emerging evidence also shows that several gut microbiome metabolites, such as SCFAs, secondary BA, indole derivatives, and amines, are directly and indirectly involved in bone metabolism, although their mechanisms of action remain to be elucidated (Figure 2).

In a proportion of UC patients, and more so in those with marked disease activity, dietary counseling may prove insufficient to provide the daily requirements of vitamins and micronutrients needed for bone health.

Vitamin D supplementation in association with adequate calcium intake should thus be prescribed to all IBD patients in whom dietary intake is below the RDA and in those requiring systemic corticosteroids [149,150]. Daily vitamin D (800–1000 IU) and calcium (800–1000 mg) supplements are advisable. Supplementation is also advisable in those UC patients in whom dietary vitamin K intake is low. A daily intake of 90–140 micrograms is considered adequate. This can be provided by supplementing commercially available formulations of vitamins K1, K2, or K3.

Further studies are needed to define whether an adequate intake of vitamins D and K, besides reducing the risk of osteopenia and osteoporosis, will prove useful for the control of chronic inflammation in UC patients.

## Data Availability

Not applicable.

## Abbreviations

The following abbreviations are used in this manuscript:

IBD	inflammatory bowel disease
UC	ulcerative colitis
BMD	bone mineral density
MeSH	Medical Subject Headings
CD	Crohn’s disease
RANKL	receptor activator of nuclear factor kB ligand
OPG	osteoprotegerin
TNF-α	tumor necrosis factor α
IPAA	ileal pouch anal anastomosis
RDA	recommended daily allowance
PTH	parathyroid hormone
VDR	vitamin D receptor
PT	prothrombin time
PIVKA-II	protein induced by vitamin K absence-II
ucOC	undercarboxylated osteocalcin
SCFA	short-chain fatty acid
BA	secondary bile acid
